# Assessment of hypertension chronic care model: Pacic application in Bosnia and Herzegovina

**DOI:** 10.1371/journal.pone.0202250

**Published:** 2018-08-14

**Authors:** Natasa Pilipovic-Broceta, Nadja Vasiljevic, Jelena Marinkovic, Nevena Todorovic, Janko Jankovic, Irena Ostric, Dimitra Kalimanovska-Ostric, Maja Racic

**Affiliations:** 1 Family Medicine Department, Faculty of Medicine, University of Banja Luka, Banja Luka, Bosnia and Herzegovina; 2 Family Medicine Teaching Centre, Primary Health Care Banja Luka, Bosnia and Herzegovina; 3 Institute of Hygiene and Medical Ecology, Faculty of Medicine, University of Belgrade, Belgrade, Serbia; 4 Institute of Medical Statistics and Informatics, Faculty of Medicine, University of Belgrade, Belgrade, Serbia; 5 Institute of Social Medicine, Faculty of Medicine, University of Belgrade, Belgrade, Serbia; 6 Faculty of Medicine, University of Belgrade, Belgrade, Serbia; 7 Institute for Cardiovascular Diseases, Clinical Center of Serbia, Belgrade, Serbia; 8 Department for Primary Health Care and Public Health, Faculty of Medicine, University of East Sarajevo, Foca, Bosnia and Herzegovina; Indiana University, UNITED STATES

## Abstract

The objectives of this study were to evaluate patients’ attitudes towards hypertension treatment according to the chronic care model and to assess the implementation of hypertension clinical guidelines in family medicine. The cross-sectional study was carried out in two randomly selected primary health care centers (Bijeljina and Prijedor), respectively in Bosnia and Herzegovina, covering the period between March and April 2016. This study sample consists of 791 respondents with hypertension purposing to measure specific actions and quality of care for hypertensive patients. The Patient Assessment of Chronic Illness Care (PACIC) was used. Treatment for the indicators of hypertension was assessed by analyzing patients' medical charts according to the recommendations of clinical guidelines. More than half of the evaluated indicators of treatment for hypertension were documented in medical charts of 84.07% patients. The average overall PACIC score was 4.18 (SD 0.59), being an average of the separate scores of 4.19 (SD 0.57) in men and 4.17 (SD 0.60) in women. Subscale means of PACIC were as follows: patient activation 4.33, delivery system design 4.36; goal setting 4.03; problem solving 4.51; follow-up and co-ordination 3.67. No statistically significant correlations in the overall score and subscale scores were found by demographic characteristics. Non-smokers had a significantly higher overall score compared to smokers (p = 0.001). As implementation of the guidelines became stronger, the reported PACIC scores rose. Continuing the education of patients in order to achieve better health care outcomes is imperative.

## Introduction

Primary health care (PHC), oriented toward family medicine (FM) model, is the point of first contact between the patients and health system, capacitated to resolve a minimum of 80% of all health problems [1]. To strengthen the PHC, the Republic of Srpska (RS), one of two entities in Bosnia and Herzegovina implemented health care reform20 years ago,in accordance with the PHC Strategy. The reform was carried out due to inconsistent delivery of health care services, unequal access to health care, inadequate financing of health care, lack of human resources and increasing percentage of elderly population[2]. To enable family physicians and family medicine nurses to become the gate keepers of health care system and provide development of recommended primary care structure dimensions, such as continuity, accessibility, comprehensiveness and coordination, new scope of services (health promotion, disease prevention, patient education and extended diagnostic and therapeutic procedures) was introduced [3].

According to World Health Organization, the probability of dying between 30 and 70 years of age from the 4 main mass non-communicable diseases (NCDs) in Bosnia and Herzegovina (BiH) is 18%, including arterial hypertension -found in 43.2% of adult people [4]. The burden of hypertension is continuously increasing and has substantial negative influence on population level [5]. Apart from the pharmacological treatment [6–8], individual behaviors, healthy diet, physically activity, treatment adherence, and regular medical appointments play a major role in hypertension control [9,10]. Previous studies have shown that the integrated, chronic care models are effective in lowering blood pressure and improving patients’ health [11–18]. The Chronic Care Model (CCM) is a framework for organizing and improving chronic illness care, based on a proactive, planned approach that incorporates patient self-care, provider, and system level interventions [19]. Application of the CCM to hypertension improves the diagnosis and management of hypertension [20].

Patients with arterial hypertension in BiH are mainly treated by family physicians. Therefore, implementation of the updated, patient-centered, evidence-based clinical guidelines in family medicine may have significant impact on the effectiveness of antihypertensive treatment and CCM [11]. Guidelines for hypertension management provide standards of care for managing hypertension; algorithms that address medication selectionand strategies for improving treatment adherence [21,22]. Several instruments have been developed to evaluate the effects of CCM implementation on care and treatment outcomes.The Patient Assessment of Chronic Illness Care (PACIC) questionnaire is the most frequently used instrument to evaluate the delivery of care for hypertensive patients [23]. It was validated in a sample of patients with hypertension, diabetes, and chronic pulmonary disease in BiH [24].

The objectives of this study were: (a) to evaluate patients’ attitudes towards treatment for hypertension according to chronic care model, (b) to assess the implementation of the clinical guideline for hypertension in family medicine, and (c) to assess the relationship between PACIC score and the implementation of clinical guideline for hypertension in family medicine. The study tests the hypothesis that implementation of clinical guidelines in family medicine is significantly associated with the patients’ assessment of the quality of hypertension care.

## Materials and methods

### Study participants

The cross-sectional study included a convience sample of 66 family medicine practices from 2 primary health care centers, Bijeljina and Prijedor. The practices were stratified by location to accurately represent the characteristics of family medicine practice in Bosnia and Herzegovina. All FPs were informed in detail by principal investigator about the research objectives and asked to sign a consent form.

Principal investigator chose a systematic, disproportionate sample of the 12 or 13 participants, from a register of hypertensive patients available to every family medicine practice, who met the inclusion criteria and agreed to be enrolled in the study. The sample size calculation was conducted to estimate the minimum number of responses. With a population size allowing for an error margin of 5% with a 95% Confidence Interval (CI), the minimum required number of responses was found to be 810. Inclusion criteria were diagnosis of hypertension according to the criteria of European Society of Cardiology (ESC) [22], over 18 years and being registered at the same family practice for at least one year. To ensure group homogeneity, patients with complications (stroke, ischemic heart disease, arterial fibrillation and heart failure, renal failure), diabetes, cognitive impairments and any other conditions which would render the patient ineligible for the study were excluded from the study.

### Measures

To measure specific actions and quality of care for hypertensive patients, the Patient Assessment of Chronic Illness Care (PACIC) was used. PACIC assesses patient-centered chronic illness care, provision of care and the capacity of self-care.As described in detail previously, itis a a multidimensional, patient centered, cognitively complex 20-item questionnaire divided into five subscales:patient activation (3 items), delivery system/practice design (3 items), goal setting/tailoring (5 items), problem solving/contextual (4 items), and follow up/coordination (5 items). Responses were rated according to a 5 point Likert-type scale ((ranging from 1 - “almost never” to 5 - “almost always”). High scores indicated a more frequent occurrence of the pertinent aspect of chronic care and perception of chronic care delivery.The study participants were scheduled to see their physician on specific date and asked to answer questions about the delivery of care in the previous 6 months or to describe the last visit. No participant identifiers were collected, so anonymity and confidentiality of the responses was ensured throughout the study. The participation in the study was voluntary, no incentives were provided.

### Demographic and clinical characteristics

A standardized questionnaire was used to collect data regarding the patients’ characteristics such as gender, age, education, marital status, place of residence, employment status, personal history, smoking status and family history (cardiovascular diseases, diabetes mellitus, stroke).

To assess the implementation of clinical guidelines for hypertension in family medicine, the indicators were selected according to the ECS [22] and national clinical guidelinefor arterial [25] what has been described elsewhere [24]. All aspects of the electronic medical record data were included in the analysis in the search for evidence of quality indicators.

Clinical data and laboratory parameters were assessedat the time of enrollment. Laboratory tests included: cholesterol, LDL-cholesterol, triglycerides, creatinine, glucose, urine protein analysis and hemoglobin, and clinical measures: body mass index (BMI) and waist circumference. Type of treatment and lifestyle counselling were registered.

The study was conducted in accordance with the World Medical Association Declaration of Helsinki of 1964, and approved by local authorities and local Bioethics Committees (BC) in participating primary health care centers (BC Prijedor: Approval No. 01-1545-3/15 and BC Bijeljina: Approval No. 6372/15).

### Statistical analysis

Socio-demographic and clinical characteristics are presented by mean values and standard deviation. Categorical variables were presented with counts and percentages while continuous ones with means and standard deviations. The significant differences between patient subgroups were divided according to demographic, socio-economic and clinical variables. PACIC scores and its five subscales were tested using the one-way ANOVA or t-test, related either to the number of subgroups, or to their non-parametric alternatives. All P values are based on two-tailed tests, and P < 0.05 was considered significant. Data were analyzed using SPSS 21 software (SPSS Inc, Chicago, III).

## Results

The study sample included of 791 patients accepting an interview out of 810 who were invited to participate in both PHC centers (response rate = 97.65%). The mean age was 63.3 years (SD = 10.32). Participants were mostly female (61.7%). Majority of patients had a secondary education level (45.5%), lived with a partner (70.3%), and were retired (38.3%). A majority of the patients (57.3%) lived in urban or suburban areas, 84.9% were non-smokers, and 40.7% were overweight. Out of 785 people that answered the question, 'What do you think about your health?',14.6% of the participants thought they had excellent health. 289 (36.8%) had good health, and 381 (48.5%) had bad health. The number of patients visiting their family doctor one to four times during the last twelve months was slightly over-represented (37.2%) (Table 1).

**Table 1:** Socio-demographic characteristics of respondents.

Characteristic	N	%
**Age groups, years**		
< 55	151	19.3
55–64	259	33.2
65+	371	47.5
**Gender**		
Male	303	38.3
Female	488	61.7
**Marital status**		
With partner	568	72.1
Without partner	220	27.9
**Education level**		
Primary	325	42.0
Secondary	352	45.5
Tertiary	96	12.4
**Employment**		
Employed	138	17.5
Non—employed	159	20.2
Retired	302	38.3
Student	4	0.5
Housewife	167	21.2
Other	19	2.4
**Place of residence**		
Urban	291	36.8
Semi–urban	162	20.5
Rural	337	42.7
**Smoking status**		
No	665	84.9
Yes	118	15.1
**BMI, kg/m**^**2**^		
Normal /(18.50–24.99)	175	23.3
>25 (overweight)	306	40.7
>30 (obese)	271	36.0
**Self reported health**		
Excellent	115	14.6
Good	289	36.8
Bad	381	48.5
**Number of patient visits**		
None	17	2.2
Once to four times	292	37.2
Five to ten times	277	35.2
More than ten times	200	25.4

BMI: body mass index

More than half of the indicators of hypertension treatment based on the clinical guideline were documented in medical charts by 84.07% of patients. The study showed that from 50% to 74.99% of indicators were documented in 45% of the patients’ medical charts, and 75% or more of the indicators were documented in 39.06% of patients medical charts. Creatinin clearance was the only one indicator not documented in medical charts of the study sample. Out of all evidenced indicators, 349 (44.12%) patients had 50% or more normal findings (Table 2). There was the statistical significant association between the number of patient visits to the family doctor and the percentage of documented indicators (p = 0.004), but there was no statistically significant association between the number of patient visits to the family doctor and the percentage of normal findings (p = 0.387).

**Table 2:** The relationship between patient characteristics and hypertension guideline indicators.

Patient characteristics		Indicators registered in medical charts (percent)											Normal indicator findings (percent)									
		<25		25–49.99			50–74.99		≥75		Total		<25		25–49.99		50–74.99		≥75		Total	
**Gender**																						
**Male**	N (%)	25	(54.3)	27		(33.8)	10	(36.)	121	(39.2)	303	(38.3)	40	(43.0)	138	(39.5)	122	(35.7)	3	(42.9)	303	(38.3)
**Female**	N (%)	21	(45.7)	53		(66.3)	226	(63.5)	188	(60.8)	488	(61.7)	53	(57.0)	211	(60.5)	220	(64.3)	4	(57.1)	488	(61.7)
**Total**	N (%)	46	(100)	80		(100)	356	(100)	309	(100)	791	(100)	93	(100)	349	(100)	342	(100)	7	(100)	791	(100)
**Age**	Mean (SD)	63.8	(14.2)	64.7		(10.3)	627	(10.5)	635	(9.5)	63.3	(10.3)	65.7	(11.5)	63.1	(10.8)	62.8	(9.4)	66.1	(12.7)	63.3	(10.3)
**<55**	N (%)	11	(25.0)	14		(17.9)	78	(22.3)	48	(15.5)	151	(19.3)	16	(17.8)	72	(21.0)	62	(18.2)	1	(14.3)	151	(19.3)
**55–64**	N (%)	6	(13.6)	21		(26.9)	107	(30.6)	125	(40.5)	259	(33.2)	14	(15.6)	109	(31.8)	134	(39.3)	2	(28.6)	259	(33.2)
**≥65**	N (%)	27	(61.4)	43		(55.1)	165	(47.1)	136	(44.0)	371	(47.5)	60	(66.7)	162	(47.2)	145	(42.5)	4	(57.1)	371	(47.5)
Total	N (%)	44	(100)	78		(100)	350	(100)	309	(100.0)	781	(100)	90	(100)	343	(100)	341	(100)	7	(100)	781	(100)
**Smoking status**																						
**No**	N (%)	43	(93.5)		69	(87.3)	294	(83.8)	259	(84.4)	665	(84.9)	81	(88.0)	297	(86.1)	280	(82.6)	7	(100.0)	665	(84.9)
**Yes**	N (%)	3	(6.5)		10	(12.7)	57	(16.2)	48	(15.6)	118	(15.1)	11	(12.0)	48	(13.9)	59	(17.4)	0	(0.0)	118	(15.1)
**Total**	N (%)	46	(100)		79	(100)	351	(100)	307	(100)	783	(100)	92	(100)	345	(100)	339	(100)	7	(100)	783	(100)
**Self-reported health**																						
**Excellent**	N (%)	8	(17.4)		4	(5.1)	49	(13.9)	54	(17.5)	115	(14.6)	11	(12.0)	40	(11.6)	63	(18.5)	1	(14.3)	115	(14.6)
**Good**	N (%)	15	(32.6)		44	(55.7)	132	(37.5)	98	(31.8)	289	(36.8)	42	(45.7)	138	(39.9)	106	(31.2)	3	(42.9)	289	(36.8)
**Bad**	N (%)	23	(50.0)		31	(39.2)	171	(48.6)	156	(50.6)	381	(48.5)	39	(42.4)	168	(48.6)	171	(50.3)	3	(42.9)	381	(48.5)
**Total**	N (%)	46	(100)		79	(100)	352	(100)	308	(100)	785	(100)	92	(100)	346	(100)	340	(100)	7	(100)	785	(100)
**Number of visits to Family physicians**																						
**None**	N (%)	1	(2.2)		4	(5.0)	10	(2.8)	2	(0.7)	17	(2.2)	0	(0.0)	11	(3.2)	6	(1.8)	0	(0.0)	17	(2.2)
**0–4**	N (%)	19	(41.3)		39	(48.8)	130	(36.8)	104	(33.9)	292	(37.2)	39	(41.9)	128	(36.9)	123	(36.3)	2	(28.6)	292	(37.2)
**5–10**	N (%)	8	(17.4)		24	(30.0)	121	(34.3)	124	(40.4)	277	(35.2)	25	(26.9)	123	(35.4)	125	(36.9)	4	(57.1)	277	(35.2)
**>10**	N (%)	18	(39.1)		13	(16.3)	92	(26.1)	77	(25.1)	200	(25.4)	29	(31.2)	85	(24.5)	85	(25.1)	1	(14.3)	200	(25.4)
**Total**	N (%)	46	(100)		80	(100)	353	(100)	307	(100)	786	(100)	93	(100)	347	(100)	339	(100)	7	(100)	786	(100)

The average overall PACIC score was 4.18 (SD 0.59), 4.19 (SD 0.57) in men and 4.17 (SD 0.60) in women. Subscale means of PACIC were as follows: patient activation 4.33, delivery system design 4.36; goal setting 4.03; problem solving 4.51; follow-up and co-ordination 3.67 (Table 3).

**Table 3:** PACIC overall and subscales scores in patients with hypertension in family medicine.

Patient characteristics	PACIC (Q1-Q20) Mean (SD)	PACIC subscales				
		Patient activations (Q1-Q3) Mean (SD)	Delivery system design/decision support (Q4-Q6) Mean (SD)	Goal setting/tailoring (Q7-Q11) Mean (SD)	Problem solving/contextual (Q12-Q15) Mean (SD)	Follow-up/coordination (Q16-Q20) Mean (SD)
**Guidelines implementation**						
**% of implemented examination, categories**						
< 25.00	3.96(0.76)	4.20(0.90)	4.02(0.79)	3.84 (0.95)	4.43 (0.77)	3.54 (1.01)
25.00–49.99	3.93(0.53)	4.13(0.71)	4.00(0.64)	3.73 (0.70)	4.27 (0.67)	3.48(0.84)
50.00–74.99	4.17(0.58)	4.29(0.76)	4.35(0.66)	4.00 (0.82)	4.51 (0.53)	3.65 (0. 89)
≥ 75.00	4.28(0.56)	4.43(0.66)	4.50(0.59)	4.15(0.76)	4.57 (0.55)	3.76 (0.87)
P-value^a^	0.001	0.002	0.001	0.001	0.001	0.049
**% of normal examination findings, categories**						
< 25.00	3.95(0.65)	4.17(0.83)	4.01(0.76)	3.84(0.79)	4.36 (0.69)	3.49 (0.89)
25.00–49.99	4.16(0.58)	4.34(0.71)	4.36(0.63)	3.98 (0.84)	4.48 (0.59)	3.69 (0.89)
50.00–74.99	4.25(0.56)	4.36(0.72)	4.44(0.63)	4.11 (0.75)	4.56 (0.52)	3.70 (0.89)
≥ 75.00	4.34(0.69)	4.43(0.63)	4.48(0.60)	4.00 (0.98)	4.64 (0.40)	3.91 (1.03)
P-value^a^	0.001	0.145	0.001	0.021	0.013	0.191
**Smoking status**						
no	4.20(0.58)	4.36(0.72)	4.38(0.65)	4.06 (0.81)	4.53 (0.57)	3.72 (0.88)
yes	4.00(0.58)	4.16(0.76)	4.22(0.70)	3.85(0.78)	4.37 (0.59)	3.44 (0.89)
P-value^b^	0.001	0.010	0.026	0.010	0.009	0.002
Self–reported health						
Excellent	4.32 (0.52)	4.43 (0.07)	4.54 (0.05)	4.22 (0.74)	4.54 (0.51)	3.90 (0.85)
Good	4.17(0.06)	4.34 (0.70)	4.35(0.59)	4.04 (0.78)	4.51(0.52)	3.69(0.87)
Bad	4.14 (0.62)	4.29 (0.76)	4.30 (0.72)	3.95 (0.83)	4.49(0.63)	3.59(0.91)
P-value^b^	0.009	0.144	0.003	0.008	0.700	0.005
Blood pressure						
Systolic						
>140 mmHg	4.02 (0.65)	4.21 (0.80)	4.17 (0.74)	3.83 (0.872)	4.42 (0.71)	3.43 (0.97)
<140 mmHg	4.22 (0.55)	4.37 (0.70)	4.42 (0.63)	4.87 (0.77)	4.53(0.53)	3.74 (0.85)
P-value^b^	0.001	0.018	0.001	0.001	0.075	0.001
Diastolic						
>90 mmHg	3.88 (0.67)	4.09 (0.97)	4.10 (0.83)	3.60(0.79)	4.34(0.67)	3.23 (0.92)
< 90 mmHg	4.21 (0.568)	4.35 (0.69)	4.39 (0.64)	4.07 (0.79)	4.52 (0.57)	3.71 (0.88)
P-value^b^	0.001	0.039	0.011	0.001	0.143	0.001

^a^ ANOVA test

^b^Student t-test

No statistically significant correlation in overall score and subscale scores were found according to age, sex, marital status, education level, place of residence, employment status and BMI categories. Non-smokers had the significantly higher overall score as well as all subscale scores (p<0.05). Patients with an excellent health reported statistically significantly higher overall score in comparison with those with self-perceived bad health (p = 0.009). There was significant association between excellent health with subscale scores, “Delivery system design/decision support” (p = 0.003), “Goal setting/tailoring” (p = 0.008) and “Follow-up/coordination” (p = 0.005). Patients with the highest percentage of implemented examination and clinical indicators (≥75%) had significantly higher overall scores (p = 0.001), in “Delivery system design/decision support” (p = 0.001), in “Goal setting/tailoring” (p = 0.001), in “Problem solving/contextual” score (p = 0.001), and in “Patient activation” (p = 0.002). There was a statistically significant difference in overall score as well as four subscale scores by blood pressure values (p<0.05). “Problem solving/contextual” score was not statistically different between patients with normal and abnormal systolic (p = 0.075) and diastolic (p = 0.143) blood pressure (Table 3).

Means of the overall PACIC scores were compared by clinical parameters using the Student t test. Patients with normal values of creatinine and waist circumference had significantly higher overall scores. No statistically significant differences were found regarding PACIC score between the patients with abnormal and normal values of glucose, cholesterol, triglycerides, proteinuria and BMI (Table 4).

**Table 4:** Differences in PACIC scores according to the values of clinical parameters.

Parameter		Number of participants with registered value	PACIC		p^a^
			Mean	SD	
Serum glucose	>6.5 mmol/	160	4.22	0.52	0.597
	<6.5 mmol/l	524	4.19	0.59	
Cholesterol	>5 mmol/l	425	4.19	0.59	0.490
	<5 mmol/l	238	4.22	0.52	
HDL- cholesterol	< 1.2 F; 1 M mmol/l	186	4.28	0.53	0.305
	> 1.2 F;1 M mmol/l	114	4.35	0.58	
LDL-cholesterol	>3.4 mmol/l	287	4.27	0.56	0.648
	<3.4 mmol/l	253	4.25	0.55	
Triglycerides	>1.7 mmol/l	331	4.22	0.566	0.388
	<1.7 mmol/l	330	4.18	0.574	
Hemoglobin	<120 F, 140 M g/l	174	4.27	0.523	0.129
	>120 F, 140 M	497	4.19	0.593	
Creatinine	<44 F, 62 M μmol/l	161	4.07	0.607	0.000
	>80 F,106 M μmol/l	464	4.28	0.551	
Proteinuria	>150 mg/24h	25	4.04	0.721	0.638
	<150 mg/24h	117	4.11	0.597	
BMI	>25 kg/m^2^	269	4.18	0.584	0.611
	<25 kg/m^2^	476	4.16	0.588	
Waist circumference (cm)	> 88 F, 102 M	195	4.22	0.533	0.004
	< 88 F, 102 M	226	4.37	0.502	

BMI: Body Mass Index; F: female; M: male

^a^Student t-test

Advice on regular physical activity was recorded in 672 (85%) and diet related counseling in 712 (90%) medical records in previous 12 months. Majority of patients used either ACE inhibitors (47.2%) or combinations of ACE inhibitors with diuretics (40.5%) (Table 5).

**Table 5:** Antihypertensive therapy.

Medication class	n	%	95%CI
ACE inhibitors	373	47.2	43.4–51.0
Combinations ACE inhibitors/diuretics	320	40.5	36.7–44.3
Calcium channel blockers	252	31.9	28.3–35.5
Beta blockers	144	18.3	15.3–21.3
Diuretics	71	9.0	6.8–11.2

95%CI: confidence interval

## Discussion

FM doctors in RS are obliged to implement clinical guidelines in the treatment of patients with hypertension. Ministry of health and social welfare in RS published clinical guidelines for the most common chronic diseases in RS in 2004. Furthermore, guidelines have been updated in 2010 [25]. The RS Agency for Certification, Accreditation and Health Care Improvement monitors and improves health care quality including implementation of clinical guidelines in family medicine. Analyzing the level of guideline implementation, it is possible to assess if the health care in FM is in accordance with chronic care model (CCM). Our intention was to assess the delivery of CCM activities in RS using patients’ medical charts and the PACIC questionnaire. The mean value of the overall PACIC of 4.18 obtained in our study was higher when compared with similar studies conducted in United Kingdom, Romania, and Germany [17–28]. It indicates, that, on average, the chronic care model has occurred “most of the time” in our society. Petersen reported that total PACIC score of 2.4 was associated with “real life scenarios” in Germany where patients with multiple chronic conditions had complex needs [28]. Al Momen reported much lower total PACIC score (1.9863) as a consequence of low patient doctor relationship in Riyadh [29]. Our findings did not show statistical significant correlation in PACIC scores according to age, sex, marital status, education level, place of residence, employment status and BMI categories. It is contrary to the original validation study scores that were positively associated with gender and the number of chronic diseases [23]. Findings of the study in India showed that PACIC 20 was slightly correlated with age, gender and chronic diseases [30]. A Swiss study showed the significant correlation between the French version of the PACIC and age [31]. Similar to our findings, in a Romanian study, age and the number of chronic conditions were not significantly associated with PACIC scores [27].

This study revealed that PACIC scores were positively related to the number of patients’ visits to FD. Patients with the highest percentages of implemented examinations and clinical indicators (≥75%) had significantly higher overall scores. “Delivery system design/decision support” being, “Goal setting/tailoring”, “Problem solving/contextual” score and “Patient activation”, There was no statistically significant difference between groups related to the percentage of clinical indicators and “Follow-up/coordination” scores. The mean score in Germany was highest for the subscale 'Delivery system design/decision support' and contrary to our findings, the lowest for 'Goal setting/tailoring' [28]. PACIC score in China was associated with the utilization of community health centers, where patients’ trusting in community health centers depended on the quality of chronic care [32]. Our study results were similar to Petersen et al. [28] and Ludt et al. revealing that higher PACIC scores were associated with more frequent FD contacts [33]. There was a statistical significant correlation between patients with normal blood pressure and overall score as well as four subscale scores. “Problem solving/contextual” score was not statistically significantly different between patients with normal and abnormal blood pressure. There was statistical significant correlation between the number of patient visits to the family doctor and the percentage of documented indicators. Frequent visits make an opportunity for developing better communication between FDs and patients in order to implement clinical guidelines and increase the quality of hypertension care in PHC centers (Table 3). Correlation between the number of patients’ visits to FD and percentage of normal findings was not statistically significant in our study. There are a lot of factors influencing normal findings such as medical treatment, lifestyle and education of patients. The role of FD is important in patients’ education on diet, physical activity as well as smoking cessation.

Similar to Krucien et al. we found the overall PACIC score significantly associated with self-reported health [34]. The majority of patients in our study are satisfied with their health status. Patients who reported excellent health had often received written recommendations on health improvement and had most of the time been asked about their goals in caring for their illness. FM teams gave a copy of the treatment plan to those patients and most of the time was considerate of the patient’s traditions.

The major strength of this study is that it is the first conducted study that has captured information in our society on the patient’s experience of hypertension care. Outcomes of the study have indicated the quality of hypertension care.

In spite of these results, there were several limitations. A majority of patients had a good, long-term relationship with their family physician, so patients may have had prior positive attitudes. The study was conducted in two primary health care centers, one in the western part and another in the eastern part of RS. Further studies including other primary health care centers in Bosnia and Herzegovina are needed to explore factors that may mediate the health care of patients, such as reform of primary health care and improvements in a field of family medicine education.

## Conclusions

Implementation of clinical guidelines significantly improves patients’ assessment of the quality of care (S2 and S3 Tables). Guidelines must be actively integrated into family practice to reach optimal outcomes. The chronic care model could be an ideal, multidimensional systemic and integrated approach to optimise all components of hypertension management. Further studies are required to identify elements that might enhance the utilization and application of clinical guidelines for arterial hypertension among family physicians [35].
